# Supplementary material for: Analysis of rotational grazing management for sheep in mixed grassland
Source: PeerJ. 2024 May 30;12:e17453. doi: 10.7717/peerj.17453 (PMC11144397; doi:10.7717/peerj.17453)
Supplement: Supplemental Information 2 [file peerj-12-17453-s002.docx]

Supplementary Material

**Table S1 DMI for sheep statistics in each grazing zone**

| Zone No. | Periods | Replications | Biomass Decrease (g/m^2^) | DMI  (kg/ewe/d) |
| --- | --- | --- | --- | --- |
| P2 | July.6-July.10 | **1** | 100.6 | 1.12 |
|  |  | **2** | 84.5 | 0.94 |
|  |  | **3** | 170.7 | 1.90 |
| P5 | July.11-July.15 | **1** | 98.2 | 1.09 |
|  |  | **2** | 130.1 | 1.45 |
|  |  | **3** | 72.3 | 0.80 |
| P3 | July.16-July.20 | **1** | 174 | 1.93 |
|  |  | **2** | 176.1 | 1.96 |
|  |  | **3** | 136.7 | 1.52 |
| P6 | July.21-July.25 | **1** | 86.2 | 0.96 |
|  |  | **2** | 118.4 | 1.32 |
|  |  | **3** | 112 | 1.24 |
| P1 | July.26-July.30 | **1** | 167.8 | 1.86 |
|  |  | **2** | 139.7 | 1.55 |
|  |  | **3** | 115 | 1.28 |
| P4 | July.31-July.4 | **1** | 139.8 | 1.55 |
|  |  | **2** | 167.6 | 1.86 |
|  |  | **3** | 176.9 | 1.97 |

**Table S2 CP and ADF of grassland statistics in each grazing zone**

| Zone No. | Periods | Replications | CP (%) | ADF (%) |
| --- | --- | --- | --- | --- |
| P2 | July.6-July.10 | **1** | 21.62 | 29.18 |
|  |  | **2** | 20.30 | 30.28 |
|  |  | **3** | 20.60 | 29.32 |
|  |  | **4** | 18.22 | 30.14 |
| P5 | July.11-July.15 | **1** | 21.98 | 29.02 |
|  |  | **2** | 19.71 | 28.35 |
|  |  | **3** | 17.43 | 30.57 |
|  |  | **4** | 16.15 | 30.68 |
| P3 | July.16-July.20 | **1** | 21.72 | 31.78 |
|  |  | **2** | 20.36 | 31.31 |
|  |  | **3** | 18.11 | 34.19 |
|  |  | **4** | 16.52 | 33.95 |
| P6 | July.21-July.25 | **1** | 21.10 | 38.45 |
|  |  | **2** | 20.55 | 32.60 |
|  |  | **3** | 20.14 | 30.76 |
|  |  | **4** | 19.33 | 33.76 |
| P1 | July.26-July.30 | **1** | 19.78 | 31.12 |
|  |  | **2** | 19.55 | 34.36 |
|  |  | **3** | 19.32 | 35.01 |
|  |  | **4** | 17.74 | 37.02 |
| P4 | July.31-July.4 | **1** | 19.95 | 32.05 |
|  |  | **2** | 18.37 | 30.16 |
|  |  | **3** | 17.00 | 30.25 |
|  |  | **4** | 17.15 | 33.34 |

**Table S3 Sheep weight statistics of each zone**

| Sheep No. | Initial weight/(kg) | Zone P2/(kg) | Zone P5/(kg) | Zone P3/(kg) | Zone P6/(kg) | Zone P1/(kg) | Zone P4/(kg) | Weight gain/(kg) | Weight gain/d/(g) |
| --- | --- | --- | --- | --- | --- | --- | --- | --- | --- |
| 002 | 42.6 | 43 | 43 | 44.5 | 45 | 46.5 | 47 | 4.4 | 146.7 |
| 037 | 43 | 44 | 44.5 | 45 | 45 | 45.5 | 47.5 | 4.5 | 150.0 |
| 013 | 43.5 | 44 | 44.5 | 45 | 46.5 | 47.5 | 49 | 5.5 | 183.3 |
| 029 | 43.5 | 44 | 45 | 46.5 | 48.5 | 49.5 | 50.5 | 7 | 233.3 |
| 026 | 44 | 44 | 44.5 | 46 | 46.5 | 47.5 | 51.5 | 7.5 | 250.0 |
| 014 | 40 | 40.5 | 40.5 | 40.5 | 41.5 | 43 | 44 | 4 | 133.3 |
| 003 | 36.5 | 37 | 37 | 40.5 | 41 | 42 | 42 | 5.5 | 183.3 |
| 032 | 35.5 | 37 | 37 | 39.5 | 40 | 40.1 | 41.5 | 6 | 200.0 |
| 007 | 46 | 46 | 46.5 | 47.5 | 48 | 50 | 53.5 | 7.5 | 250.0 |
| 028 | 47 | 49 | 50.5 | 51 | 51.5 | 52.5 | 53.5 | 6.5 | 216.7 |
| 056 | 39 | 40 | 41.5 | 42.5 | 44 | 44.5 | 44.5 | 5.5 | 183.3 |
| 015 | 47 | 48 | 48 | 48 | 50 | 53 | 54.5 | 7.5 | 250.0 |
| 253 | 45 | 45 | 47 | 47.5 | 48.5 | 51 | 51.5 | 6.5 | 216.7 |
| 001 | 40.5 | 42 | 43 | 43.5 | 44 | 45.5 | 47 | 6.5 | 216.7 |
| 089 | 45 | 46 | 46.5 | 46.5 | 47.5 | 47.5 | 49.5 | 4.5 | 150.0 |
| 212 | 41 | 42 | 43.5 | 45 | 45 | 45 | 45 | 4 | 133.3 |
| Average | 42.44 | 43.22 | 43.91 | 44.94 | 45.78 | 46.91 | 48.25 | 5.81 | 193.3 |
